# Semi-automated 3D Leaf Reconstruction and Analysis of Trichome Patterning from Light Microscopic Images

**DOI:** 10.1371/journal.pcbi.1003029

**Published:** 2013-04-18

**Authors:** Henrik Failmezger, Benjamin Jaegle, Andrea Schrader, Martin Hülskamp, Achim Tresch

**Affiliations:** 1 Max Planck Institute for Plant Breeding Research, Cologne, Germany; 2 Institute for Genetics, University of Cologne, Cologne, Germany; 3 Botanical Institute III, University of Cologne, Cologne, Germany; University of California Riverside, United States of America

## Abstract

Trichomes are leaf hairs that are formed by single cells on the leaf surface. They are known to be involved in pathogen resistance. Their patterning is considered to emerge from a field of initially equivalent cells through the action of a gene regulatory network involving trichome fate promoting and inhibiting factors. For a quantitative analysis of single and double mutants or the phenotypic variation of patterns in different ecotypes, it is imperative to statistically evaluate the pattern reliably on a large number of leaves. Here we present a method that enables the analysis of trichome patterns at early developmental leaf stages and the automatic analysis of various spatial parameters. We focus on the most challenging young leaf stages that require the analysis in three dimensions, as the leaves are typically not flat. Our software TrichEratops reconstructs 3D surface models from 2D stacks of conventional light-microscope pictures. It allows the GUI-based annotation of different stages of trichome development, which can be analyzed with respect to their spatial distribution to capture trichome patterning events. We show that 3D modeling removes biases of simpler 2D models and that novel trichome patterning features increase the sensitivity for inter-accession comparisons.

## Author Summary

The patterning of trichomes (leaf hair) on the surface of a leaf is a paradigm for studying gene regulation in developmental processes. The statistical analysis of trichome patterning requires automated methods for the location of trichomes on a curved leaf surface. This is particularly challenging for young, strongly bent leaves. We have developed the TrichEratops software that reconstructs 3D leaf surfaces from 2D stacks of conventional light-microscopy pictures. TrichEratops also calculates statistical patterning features, thereby greatly facilitating the whole data acquisition process. We show, using two *Arabidopsis thaliana* mutants, that 3D modeling removes biases and increases the discriminatory power of trichome pattern analysis.

## Introduction

Leaf trichomes in *Arabidopsis thaliana* (*A. thaliana*) represent a well-studied model system for the analysis of patterning processes in plants. Leaf trichomes are single cells that are formed on young developing leaves [1]. At their first appearance trichomes are typically separated by three or more epidermal cells. Incipient trichomes undergo several rounds of endoreduplication accompanied by massive growth and branching. Concomitant with trichome differentiation, epidermal cells divide and expand resulting in a further separation of existing trichomes. At early stages of leaf development, three zones can be distinguished, the tip region containing only mature trichomes, an intermediate region with various stages of trichome development and a basal region in which trichomes are initiated. At later leaf developmental stages trichome initiation ceases and the expansion of the leaf through epidermal divisions/expansion [2] leads to the final distribution of trichomes.

The genetic and molecular analysis of trichome development has revealed models that explain trichome development by a gene regulatory network of trichome promoting and inhibiting factors. Two probably in parallel acting mechanisms have been recognized [3]. One mechanism is based on a regulatory feedback loop of the trichome promoting genes *TRANSPARENT TESTA GLABRA1* (*TTG1*), *GLABRA1* (*GL1*) and *GLABRA3* (*GL3*)/*ENHANCER OF GLABRA 3* (*EGL3*) as well as the trichome inhibiting factors *TRIPTYCHON* (*TRY*) and *CAPRICE* (*CPC*) and their close homologs [3], [4]. In this activator-inhibitor scenario the activators transcriptionally activate the inhibitors that can move into the neighboring cells where they inhibit the activity of the activators. The second mechanism explains patterning by an activator-depletion mechanism [5], [6]. Here, *TTG1* can freely move between cells and is captured by GL3 in trichome precursor cells. As a result the activator *TTG1* is not available in the immediate vicinity of trichome initials. While many aspects of these models have been experimentally validated, it becomes increasingly clear that a mechanistic understanding of trichome patterning requires a more quantitative analysis. Towards this end, it would be necessary to have a high spatial resolution of the trichome distribution on mature and young leaves.

Several approaches have been published that enable a high-resolution 3D reconstruction of mature as well as of young leaves. *Lee* and coworkers used the optical projection tomography method to create 3D reconstructions of various plant organs including leaves and showed that this method can be used for high-resolution morphological analysis in plants including trichomes on mature leaves [7]. Kaminuma and coworkers applied micro X-ray computed tomography to visualize the trichome distribution and developed a strategy to automatically recognize trichomes on mature leaves [8]. The trichome distribution on young developing leaves was studied by Confocal Laser Scanning Microscopy. Young leaves were stained with propidium iodide and stacks of confocal images were assembled to 3D images that in turn were used to extract relevant leaf structures [9], [10]. The three methods have in common that they require either instrumentations not easily available or that they are very time consuming. We therefore aimed to develop a new simple method that enables the rapid acquisition by conventional light microscopy and analysis of the trichome pattern on young and old leaves (Table S1).

The method described here addresses an essential problem. Young *A. thaliana* leaves are typically not flat but bent at the leaf edges. Thus, the biologically relevant distances are shortest paths on the leaf surface and therefore require a 3D surface reconstruction. We propose a method for the modeling of planar surfaces of microscopic objects using simple light microscopy. By continuous variation of the microscope focus, a stack of images is generated such that each point in the plane is in focus in one stack image. Focus stacking is a well known method in microscopic imaging in order to capture sharp images of 3D objects. Different methods can be used for the determination of the sharpest point in the stack [11]–[15]. We use the Sobel transform [16] as a measure of sharpness to determine for each point on the leaf its position in the image stack. We then fit an elastic map to this cloud of (x,y,z) tuples, which provides a smoothed, realistic fit of the object surface. As a convenient side product we generate a sharpened 2D image from the trichome image stack (see Methods). We have developed TrichEratops, a software that covers all steps of this analysis: It allows the user to conveniently process the series of pictures into one sharp image, and to mark trichomes on the leaves; It estimates the 3D surface and calculates, among other spatial statistical features, the geodesic distances between the trichomes. We demonstrate the utility of our software and scan the leaf surfaces of challenging young leaves of Col-0 wild type and a genotype with Col-0 background carrying a T-DNA insertion the *CPC* gene. We discriminate the two genotypes by using summary statistics of their trichome distribution.

## Results

### Creation of a 3D surface model from a stack of 2D pictures

**Figure 1:**
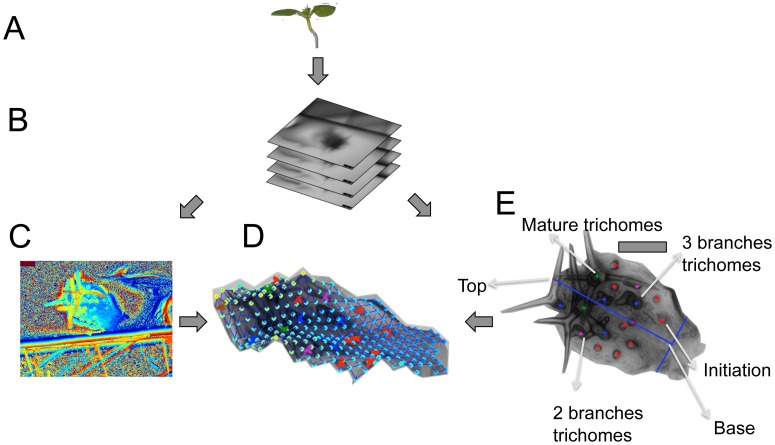
Work flow for the creation of the 3D reconstruction. A: The 3rd true leaf is dissected from a one week old seedling. B: A stack of pictures is generated by varying the focus using a conventional light microscope. C: A height map of the leaf is created by taking for every (x,y) position the z position at which the vicinity of this point appears sharpest. Height values are color-coded, ranging from low (blue, green) to high (yellow, red). D: A sharp 2D image of the leaf is generated by taking for each (x,y) position the corresponding pixel intensity in the z-stack image specified by the height map. The position of trichomes and their stages are determined in this 2D view. E: A 3D surface model of the leaf is calculated from the picture stack by fitting an elastic map to the height map, and the trichomes are mapped onto this surface. Scale bars 100 µm.

As leaves, in particular young leaves, are frequently not flat but bent the distances between trichomes cannot appropriately be measured directly by their 2D Euclidean distance on the sharpened image. The 2D Euclidean distance systematically underestimates the real, i.e. geodesic distance, of two points on a curved surface. Depending on the bending of the leaf, the difference between Euclidean and geodesic distances can be large. As leaf bending in turn varies considerably among the leaves of one *A. thaliana* line it is important to generate a 3D leaf surface model. Towards this end we dissected young leaves and created a stack of pictures by continuously varying the focus in conventional bright field microscopy (Figure 1A,B). The pictures were captured by taking a movie. Once the leaf is dissected, the 2D picture stack can be generated in a few seconds.

We used the Sobel transform to assign to each (x,y) position a z-stack position for which its vicinity in the corresponding 2D image appears sharpest. The z-axis for each stack was properly scaled by acquiring a reference of known thickness positioned next to the leaves ([17], see Figure 1C). Due to inaccuracies in z-positioning, this set of (x,y,z)-tuples merely defines a 3D cloud rather than a 2D leaf surface in 3D space. We therefore fit an elastic map (with an underlying rectangular 2D grid structure) to the cloud of (x,y,z) tuples [18]. Our elastic map is a continuous function f of the 2D coordinates (x,y). It can be viewed as a smoothing operator, which replaces the observed z position at given (x,y) coordinate by its smoothed fit z′ = f(x,y) (Figure 1D). The transformation of a 2D image stack into an elastic map takes about 5 min per leaf. The elastic map models the leaf surface as a 2D manifold, which allows us to use the geodesic distances between two points (i.e., the shortest path between them).

### Efficient annotation and analysis of trichome stages by TrichEratops

**Figure 2:**
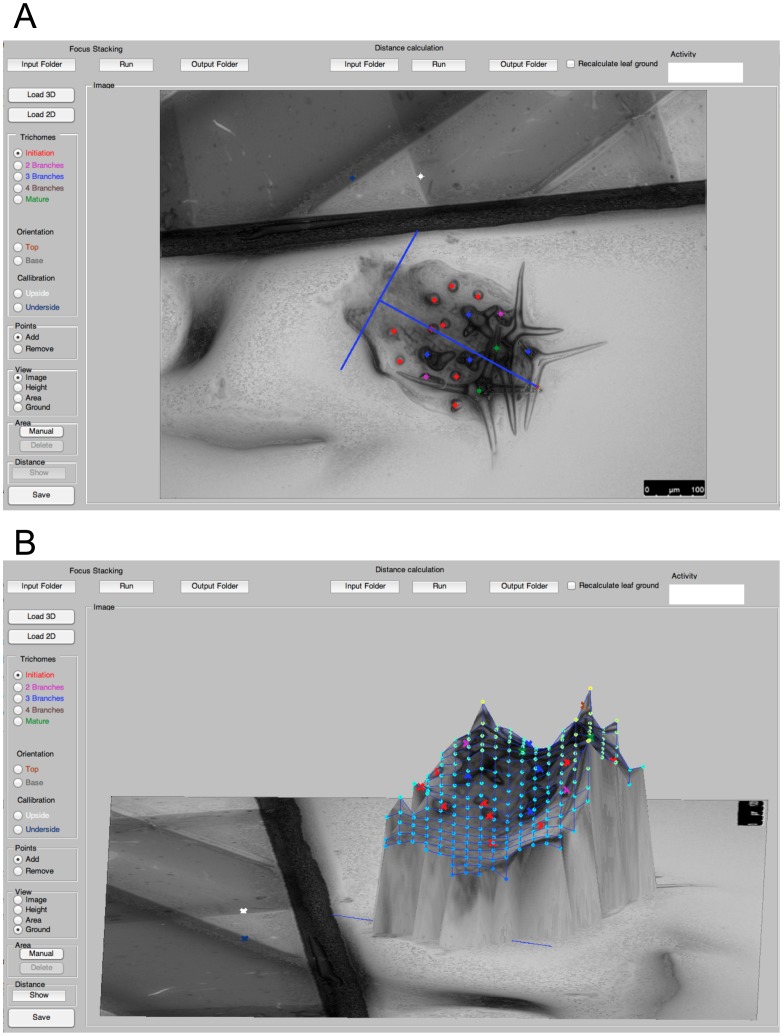
Graphical User Interface of TrichEratops. TrichEratops offers 4 different views of the leaf surface. The 2D sharpened image view (A) is used to mark trichome positions and their classes (red, purple, blue and green dots). Additionally, the leaf-centric xy-coordinate system can be set (blue axes), and the calibration marks for z-stacking can be specified (white and dark blue dot on the top half of the image). The 3D surface view (B) serves as a visual control of the reconstruction quality. The shortest paths between trichomes can be included optionally. The top control panel offers functionality for loading, saving, processing, and analysis of the image stacks.

To identify moderate changes in trichome patterning, the different trichome developmental stages within young leaves have to be acquired (average leaf length of 320 µm for Col-0 wild type). Towards this end we define four developmental classes of trichomes. For the analysis of trichome patterning, we do not discriminate between all previously defined developmental stages [19] but rather focus on four stages. The first class is formed by trichome initiation sites. This class includes all unbranched trichomes. The second and third class are trichomes with two and three branches, respectively. The last class consists of mature trichomes with a characteristic, incrusted cell wall (Figure 1E). Only leaves with one to six mature trichomes were analyzed. Together these classes define three zone: the patterning initiation zone (class 1), the differentiation zone (class 2 and 3) and the region containing the mature trichomes (class 4). TrichEratops facilitates the annotation of trichome stages on a leaf by providing a graphical user interface (Figure 2) for the marking of the four trichome classes on a sharpened 2D image of an *A. thaliana* leaf (this sharpened image is a side-product of the Sobel transform, see Methods). Since subsequent feature extraction steps critically depend on the accuracy and completeness of the trichome annotation, we did not attempt to automate this step.

**Figure 3:**
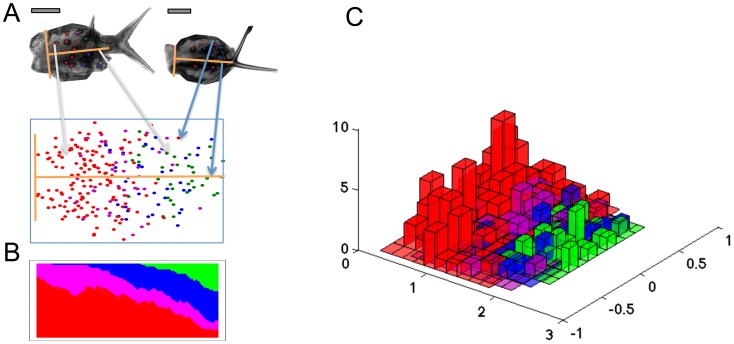
Meta leaf construction and analysis of spatial distribution of trichome classes for the Col-0 genotype. A: The meta leaf is generated by transforming all trichomes of all leaves of a given genotype to a common coordinate system (yellow axes) as illustrated by two sample leaves. The origin of each leaf is defined manually, and the unit vector in longitudinal axis direction is defined by the center of mass of the leaf. The basal axis is perpendicular to the longitudinal axis. The meta leaf shows the distribution of different trichome classes across the leaf, where red (respectively magenta, blue, green) dots indicate initiation (respectively two branch-, three branch-, and mature trichomes). B: The trichome localization along the longitudinal leaf axis is visualized. The vertical axis shows the proportion of different trichome classes at a given distance from the origin. C: The distribution of trichome classes on the meta leaf surface is shown in a 3D histogram. Trichome numbers at each position are shown as bars. The colors for the trichome classes are chosen as in A. Scale bars 100 µm.

In order to account for variation in leaf size and to obtain a unified leaf representation, we defined an orthogonal coordinate system on each leaf. The origin of the coordinate system is placed in the middle of the petiole at the leaf lamina's base. The y-axis and its scaling are determined manually by a unit vector pointing from the origin towards the top of the leaf, such that it separates the leaf into two equal halves. This implicitly defines the direction and scaling of the baseline x-axis (Figure 3A). The trichome positions of all leaves from one accession are then mapped onto a “meta leaf”, a comprehensive 2D representation of the class-specific trichome positions (Figure 3A). The meta leaf lays the basis for the calculation of relevant spatial characteristics and for their statistical comparison between different lines. The distributions of each trichome class along the basal-proximal axis are visualized in a 2D plot (Figure 3B) and in a 3D histogram showing the absolute frequencies as a function of their position on the meta leaf (Figure 3C). This type of analysis will enable a comparison of the spatial arrangement of the developmental zones between different *A. thaliana* genotypes (see Figure S5).

### Accurate extraction of trichome patterning statistics by TrichEratops

To evaluate the accuracy obtained by measuring the geodesic instead of the euclidean distances we compared the Col-0 wild type line with *cpc* mutant (*cpc-2*) known to have a higher trichome density at all leaf developmental stages tested [20].

**Figure 4:**
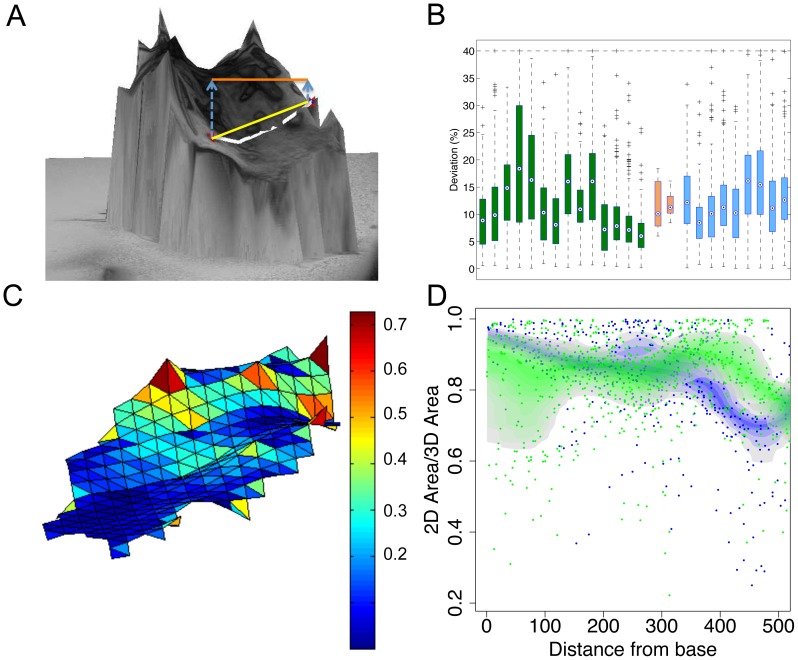
Comparison of Euclidean and geodesic distances. A: 3D reconstruction of an *A. thaliana* leaf surface. The two red dots mark the origins of two trichomes. They illustrate the difference between geodesic distance (white line), Euclidean distance with 3D coordinates (yellow line) and 2D coordinates (orange line). B: Calculation of the 2D distance relative to the geodesic distance (2D distance in percent of geodesic distance). These relative values are summarized in box plots, one for each leaf (wild type in green, *cpc-2* in blue). The orange boxes show the distribution of the median of these ratios for wild type (left) and *cpc-2* mutants (right). C: Spatial distortion (2D area divided by 3D area) of the leaf surface. We calculate the distortion for each triangle of the elastic map. Triangles are color-coded by their distortion value. D: Diagram of the 2D to 3D ratios of the triangles along the longitudinal leaf axis (green: wild type, blue: *cpc-2*). The colored bands show the 50% confidence bands for each genotype. Scale bars 100 µm.

By combining the 2D trichome position information with the elastic map representation of a leaf, we can assign to each trichome a point on the 3D leaf surface (Figure 1). This suggests three distinct possibilities to measure the distance between two trichomes: The Euclidean distance on the 2D image, the Euclidean distance in the 3D reconstruction, and the geodesic distance on the 3D reconstruction of the leaf surface (Figure 4A). We assessed the systematic differences between the 2D Euclidean distance and the geodesic distance for each pair of trichomes (Figure 4B). The median deviation of the geodesic distance to the 2D distance is 9.05% for the wild type *A. thaliana* and 11.64% for the leaves of the *cpc-2* mutant. A closer investigation reveals that both steps, the transformation from 2D to 3D Euclidean distances, and the step from 3D Euclidean distance to geodesic distance, contribute substantially to this deviation (Figure S3B and S3A). The effect of the latter step is more pronounced, which demonstrates the importance of a realistic 3D leaf representation.

Similarly, the leaf area calculations derived from 2D and 3D reconstructions can differ substantially (Figure S4). As the differences between the two calculation methods depend on the form of the particular leaf, we developed methods to visualize the local differences. We present the leaf surface as a network of triangles that are colored in blue for low differences in the leaf area and red for big differences (Figure 4C) or simply in a diagram focusing on the basal-distal axis (Figure 4D).

When comparing wild type and *cpc-2* mutants (Figure 4B), we found no indication that local distortion differs. However, *cpc-2* distortion is systematically higher than in wild type for the distal parts of the leaf. Thus, 2D distance measurements would systematically underestimate trichome distances in distal parts of the *cpc-2* mutants relative to wild type distances and therefore bias their comparison. This shows that 3D modeling is required to generate reliable and meaningful features.

### Comparison between wild type and mutant trichome patterns

To facilitate the comparison of the trichome pattern between different lines we developed several methods to quantify different aspects of the spatial distribution of trichome classes and studied the differences between wild type and *cpc-2* mutants.

**Figure 5:**
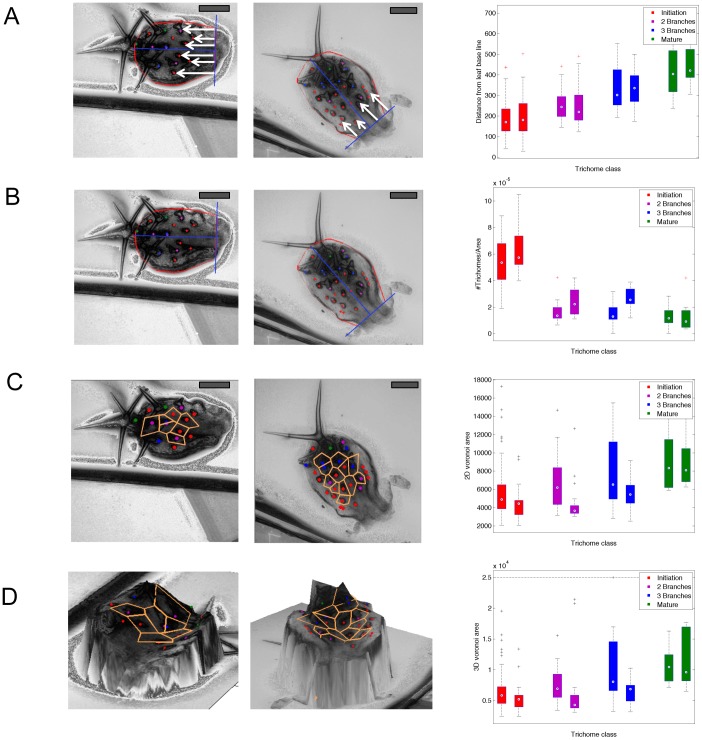
Analysis of trichome patterning by various spatial statistics. Left panel: Example of a Col-0 leaf, middle panel: Example of a *cpc-2* leaf, right panel: box plots of the statistics, stratified for trichome classes (red, magenta, blue, green) and genotypes (left box, right box). A: Distance from the leaf base line. B: Trichome density calculated as the number of trichomes divided by 3D leaf area. C: Voronoi areas of the trichomes defined by 2D Euclidean distance. D: Voronoi areas of the trichomes defined by 3D Euclidean distance. Scale bars 100 µm.

In a first step, we compared the relative proportions of trichome classes along the basal-distal axis. We found similar proportions in wild type and the *cpc-2* mutant indicating that the speed of trichome development is in the same range (Figure S1). Next, we determined the distances of trichomes from the leaf basis and compared these values separately for all trichome classes (Figure 5A). We found no significant differences between wild type and *cpc-2* mutants. Thus the spatial distributions of the zones in the leaf are similar.

The meta leaf minimizes the influence of the leaf shape and areas by normalizing it to a unit shape. This is particularly important, because the wild type has a bigger leaf index (i.e., minor axis (leaf width) divided by major axis (leaf length)) than the *cpc-2* mutant (Figure S6 C, Wilcoxon test:, p = 0.0051).

The previous finding that trichome density (defined as the number of trichomes per leaf area) in *cpc-2* mutants is increased in young and mature leaves [20] raises the question how the pattern originates. The class specific localization data enabled us to compare the trichome density for each class and thereby the developmental stages (Figure 5B). We found significant differences in the trichome densities for the three branched trichomes (Wilcoxon test, p-value<0.05, Table S3) and no significant differences in the densities of initiation trichomes and 2 branched trichomes. Note that young leaves were selected by their number of mature trichomes (strictly less than 7), making a comparison of the mature trichome density meaningless (Table S2).

There were no obvious differences in the regional distribution of trichome densities between wild type and *cpc-2* mutants (Figure 3, Figure S5). We therefore refined our analysis by calculating the Voronoi cells for all trichomes (see Methods). The Voronoi tessellation decomposes the leaf in regions, one for each trichome. Each point on the leaf surface is assigned to its proximal trichome, where distance is either measured by 2D Euclidean distance (Figure 5C) or 3D Euclidean distance (Figure 5D). Note that there are no efficient algorithms for calculating the Voronoi tessellations with respect to geodesic distance. The area of a Voronoi cell is a local measure of the inhibition strength exerted by its central trichome. The Voronoi areas of marginal trichomes are infinite and thus excluded from our analysis. The comparison of the Voronoi cell areas between the wild type and the *cpc*-2 mutant reveals that trichomes have significantly smaller Voronoi regions in the *cpc-2* mutant for the first three trichome classes (consistently, p<0.05 in a Wilcoxon test, Table S5). No statistical test was performed for the mature trichomes, for the reasons explained above. Alternatively, the geodesic distance of a trichome to its nearest neighbor can be considered as a measure of spatial inhibition. Again, we found a significant difference in geodesic distances between wild type and *cpc-2* (Wilcoxon test, p = 0.0003, Table S6).

## Discussion

Although the final trichome pattern is generated by two events, the initiation in young leaves and the separation by epidermal cell divisions and expansion, most of the genetic analysis is based on the pattern seen in mature leaves. The analysis of trichome initiation is complicated by the fact that young leaves are typically not flat. One possibility is the reconstruction of propidium iodide stained Confocal Scanning Laser Microscopy images [9], [10]. While this method has the advantage of a high resolution the generation of image stacks and subsequent analysis is time consuming. In contrast, picture acquisition with a light microscope leaves the tissue and thus avoids artifacts. To facilitate the analysis of young leaf stages and early stages of trichome initiation and -development we devised TrichEratops, a software that enables the analysis of relevant parameters on the three dimensional leaf surfaces derived from a rapidly acquired image stacks by conventional light microscopy. The actual acquisition of images takes only few seconds and TrichEratops enables a fast and convenient semi-automated analysis.

### Geodesic versus Euclidean distances

How relevant is it to calculate the geodesic rather than the Euclidean distances? When calculating the differences for individual leaves we found a 10%–20% difference between the two types of measurements (Figure 4). In the light that the differences in trichome density for each trichome class is rather small between the well-established trichome density mutant *cpc* and wild type (Figure 5), it is absolutely necessary to measure the geodesic distances when studying young leaves. Moreover, we found that the geodesic and Euclidean distances varied locally (Figure 4C). This in turn would lead to a misinterpretation of regional distances and densities depending on how much the leaves are bend.

### Analysis tools, what do we learn from which method?

For an evaluation of our analysis tools we chose the comparison of wild type with the trichome density mutant *cpc* [20]. Our analysis tools are designed to capture most relevant aspects of trichome patterning step by step.

In a first step, the whole leaf area and the leaf index are determined to detect gross variations in leaf shape that in turn may influence the trichome patterns. The second step is the creation of the meta leaf and the analysis of the trichome distribution along the longitudinal axis on a normalized leaf surface area. The distinction between different trichome developmental stages enables the detailed analysis of different aspects: 1) the relative size of the trichome initiation zone, the differentiation zone and the mature trichome zone. 2) the relative overlap of the three zones. As shown in Figure 3B there is already substantial overlap between the four trichome classes in wild type. Our finding that this is unchanged in *cpc* mutants indicates that the higher trichome density in *cpc* mutants originates during trichome initiation. The 2D presentation of the trichome distribution on a normalized leaf enables to recognize mutants in which the relative distribution on the leaf is changed as exemplified by lines overexpressing GL1 [21] in which trichome density is reduced in the mid region of the leaf.

The third step is the analysis of global and local trichome density. The global trichome density is a coarse measure, as it simply relates the number of trichomes to the leaf area. The Voronoi area is a local measure of trichome density; it measures the area around trichomes that is free of other trichomes. All peripheral trichomes are not considered and therefore border effects are excluded. Therefore the Voronoi area analysis is likely to be more suitable to capture subtle changes in the trichome initiation caused by interactions between the trichomes. The difference between the two methods is demonstrated in Figure 5B and 5D. The Voronoi areas are smallest for the initiation stage and gradually increase for more advanced stages of trichome development.

In summary, we provide a new semi-automated tool enabling the fast acquisition of image stacks, the 3D construction and analysis of relevant aspects of trichome patterning. This tool will enable a more detailed quantitative analysis of *A. thaliana* lines for a better understanding of the molecular mechanisms of trichome patterning. The combination of light microscopy and surface reconstruction has the potential of analyzing other plant material (root hair, seedlings, and flower sepal/petal). In general it could be applied for any 3D reconstruction tasks of a bent surface.

## Methods

### Plant material and growth conditions

For all experiments, the *A. thaliana* ecotype Columbia-0 and the *cpc-2* mutant [22] were grown on soil. Seeds were stratified (1 week, 4°C) and shifted to long day conditions (16 h white light, 90–120 µmol m^−2^ s^−1^/8 h darkness; 21°C).

### Microscopy

The third true leaf was dissected from one week old seedlings. Dissected leaves were transferred to a slide previously covered by 1% agar to avoid desiccation. To allow comparison between leaves we selected leaves with a maximum of 6 matures trichomes. Manual stacks of the leaves were acquired with a Leica DM5000B microscope equipped with a LEICA DFC 360 FX camera using a 10× objective. While the experimenter manually focuses along the z-axis by turning the focus of the light microscope, the Leica LAS AF software automatically generates a series of pictures ( = z-stack) by acquiring one picture per 104 ms . Start and end point were the last and first unsharp layers. 50–70 pictures were taken per leaf. To scale the Z axis the “dissector Z-axis mechanical method” was used with a piece of a cover slide (#1, art Nr H878, ROTH, Karlsruhe) positioned next to the leaf as described before (Xavier-Vidal, 2010). The thickness of the cover slide was determined using a scaling slide, PYER SGI LIMITED (1mm/0,01 mm DIV, Kent, UK), to set the exact ratio pixel/mm. For this the piece of a cover slide was placed vertically in agar to allow a precise measurement of its thickness (see Figure S2).

### Focus stacking and image sharpening

In order to get a 3D map of Z positions from the stack, the sharpest value for every pixel needed to be found. For this purpose we used the fact that clear edges indicate sharp pixel. Edges in the Images were detected by the Sobel transform [16]. A position in the image was considered as sharp if its Sobel value or the Sobel value in spatial proximity (5*5 window) reached its maximum. This resulted in a z-map of stack positions (Figure 1).

This approach was inspired by the Stack Focuser plugin for ImageJ by Michael Umorin. The z positions can be used to generate a sharpened 2D image from the image stack, by choosing for each (x,y) position the pixel intensity of the image at the corresponding z stack position (see Figure 1E).

### Surface modeling by elastic maps

Elastic maps are a tool to approximate non-linear principal manifolds [18]. Here, we use an elastic map for the robust reconstruction of the leaf ground from the Z-stack map.

Let S bet the coordinates *(x,y,z = stack position)* of the pixels in the image. The image is covered by an *n*-dimensional grid.

This grid defines a connected unordered graph *G(Y,E)* where *Y* is the collection of graphs nodes and *E* the collection between these nodes respectively. Furthermore, we define ribs, *R*, as a collection of edges between three consecutive nodes for which the bending energy is calculated.

Every data point *s* in *S* is assigned to its host node 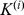, the closest node in the grid. Thus the dataset *S* is divided into sub-collections 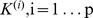:
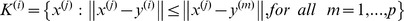
Three energies are defined for the elastic map:
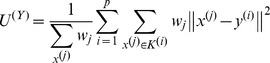


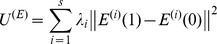


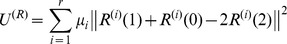
Where *U(y)* is the average weighted square between pixels and the graph nodes, *U(E)* is the energy of elastic stretching of edges in the graph and *U(R)* is the energy of elastic bending of edges in the graph.

The overall energy of the elastic map 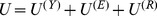 is minimized by the EM algorithm.

The overall energy of the elastic map 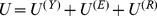 is minimized by the EM algorithm.

### Leaf detection

The leaf was identified by Otsu thresholding [23] of the grey values in the image. Large trichomes that extended over the leaf area were cut using morphological operations: Morphological opening by a disc shaped structuring element, larger than the broadest trichome was applied. This operation includes an erosion that deletes trichome structures and a deletion that rebuilds the leaf structure [24].

Some leaves were difficult to segment as they were in spatial proximity to the cover slides. For these leaves we implemented a manual segmentation in our software that enables to draw a line around the leaf and to take the area in the convex hull of this line as leaf area.

### TrichEratops

All analysis steps were implemented in a software called TrichEratops (Figure 2). This software has a Graphical User Interface, in which z-stacks are loaded and first converted into a sharp image. Trichomes can be then marked with their individual classes. A coordinate system can be drawn into the image and trichome positions are transformed to this coordinate system to ensure comparability.

From the z-stack, 3D surface and area are calculated and geodesic distance can be derived for the trichomes.

The software can handle 3D images as well as 2D images (for older leaves). All the analysis steps were implemented in Matlab. The TrichEratops software is available under the GPL3.0 license at https://sourceforge.net/projects/tricheratops/.

### Spatial statistics of trichome patterning

2D Euclidean distance is the simple vector length between two points. For the 3D Euclidean distance, the z coordinate was determined by the elastic map. For the geodesic distance the shortest path on the 3D mesh was calculated by the Fast Marching algorithm [25], [26]. The Matlab implementation of Gabriel Peyré was used for this task (http://www.mathworks.com/matlabcentral/fileexchange/6110). Voronoi diagrams are a known method to investigate biological patterning [9]. Standard Matlab methods were used for the calculation of the voronoi cells.

## Supporting Information

Figure S1 Relative abundance (in %) of trichome classes (red: initiation, magenta: 2-branch trichomes, blue: 3-branch trichomes, green: mature trichomes) for wildtype (left) and the *cpc-2* mutant leaves (right). Error bars show standard deviations.
(TIF)

Figure S2 Microscopic pictures used to scale the z-axis. A: A scaling slide PYER SGI LIMITED (1 mm/0.01 mm DIV, Kent, UK) used to scale the 2D distance of the microscope. From that the ratio pixel per µm was determined. Each graduation equals 10 µm. B: A piece of a cover slide used for the z-axis scaling was placed vertically into agar. The line shows how the thickness of the cover slide was precisely measured. Subsequently, this piece of a cover slide was placed flat next to an acquired leaf and served as a reference for the z-axis.
(TIF)

Figure S3 A: Relative increase (in %) of geodesic distances between two trichomes over the corresponding 3D Euclidean distances, summarized as separate boxplots for each leaf. Wildtype leafs are marked in green, leafs of *cpc-2* are marked in blue. The orange boxes show the distribution of the median increases for wild type (left) and *cpc-2* (right). B: Relative increase (in %) of 3D Euclidean distances between two trichomes over the corresponding 2D Euclidean distances. Coloring is as in A), identical leaves are shown in the same column as in A).
(TIF)

Figure S4 Comparison of 2D leaf area and 3D leaf area. Each line corresponds to one leaf (green: wild type, blue: *cpc-2*). The lower point of each vertical line corresponds to the 2D area, the upper point shows the 3D area. Leaf area is given in arbitrary units.
(TIF)

Figure S5 Meta leaf construction and analysis of spatial distribution of trichome classes for the *cpc-2* genotype. A: The meta leaf is generated by transforming all trichomes of all leaves of a given genotype to a common coordinate system. The meta leaf shows the distribution of different trichome classes across the leaf, where red (respectively magenta, blue, green) dots indicate initiation (respectively two branch-, three branch-, and mature trichomes). B: The trichome localization along the longitudinal leaf axis is visualized. The vertical axis shows the proportion of different trichome classes at a given distance from the origin. C: The distribution of trichome classes on the meta leaf surface is shown in a 3D histogram. Trichome numbers at each position are shown as bars.
(TIF)

Figure S6 Comparison of leaf area, leaf length and leaf index for Col-0 and *cpc-2*. A: Leaf Area, given by the surface area of the elastic map. B: Leaf length, distance between leaf top and leaf base point. C: Leaf index, given by the quotient of leaf minor axis and leaf major axis.
(TIF)

Table S1 Comparison between TrichEratops and other existing methods. Despite of all other methods TrichEratops combines light microscopy (without long sample preparation) and 3D reconstruction of the leaf surface. Furthermore it calculates patterning features similar to existing methods (voronoi area, metaleaf, coordinates).
(DOCX)

Table S2 Trichome counts for both genotypes and counts of finite Voronoi cells. Finite Voronoi cells are Voronoi regions that are bounded and whose edge points lie on the leaf surface.
(DOCX)

Table S3 Wilcoxon test for difference in trichome density between Col-0 and *cpc-2*.
(DOCX)

Table S4 Wilcoxon test for difference in 2D Voronoi Area between Col-0 and *cpc-2*.
(DOCX)

Table S5 Wilcoxon test for difference in 3D Voronoi Area between Col-0 and *cpc-2*.
(DOCX)

Table S6 Wilcoxon test for difference in geodesic distance to nearest neighbor between Col-0 and *cpc-2*.
(DOCX)

Text S1 Supplementary References.
(DOCX)
