# Supplementary figures and images for: Semi-automated 3D Leaf Reconstruction and Analysis of Trichome Patterning from Light Microscopic Images
Source: PLoS Comput Biol. 2013 Apr 18;9(4):e1003029. doi: 10.1371/journal.pcbi.1003029 (PMC3630213; doi:10.1371/journal.pcbi.1003029)

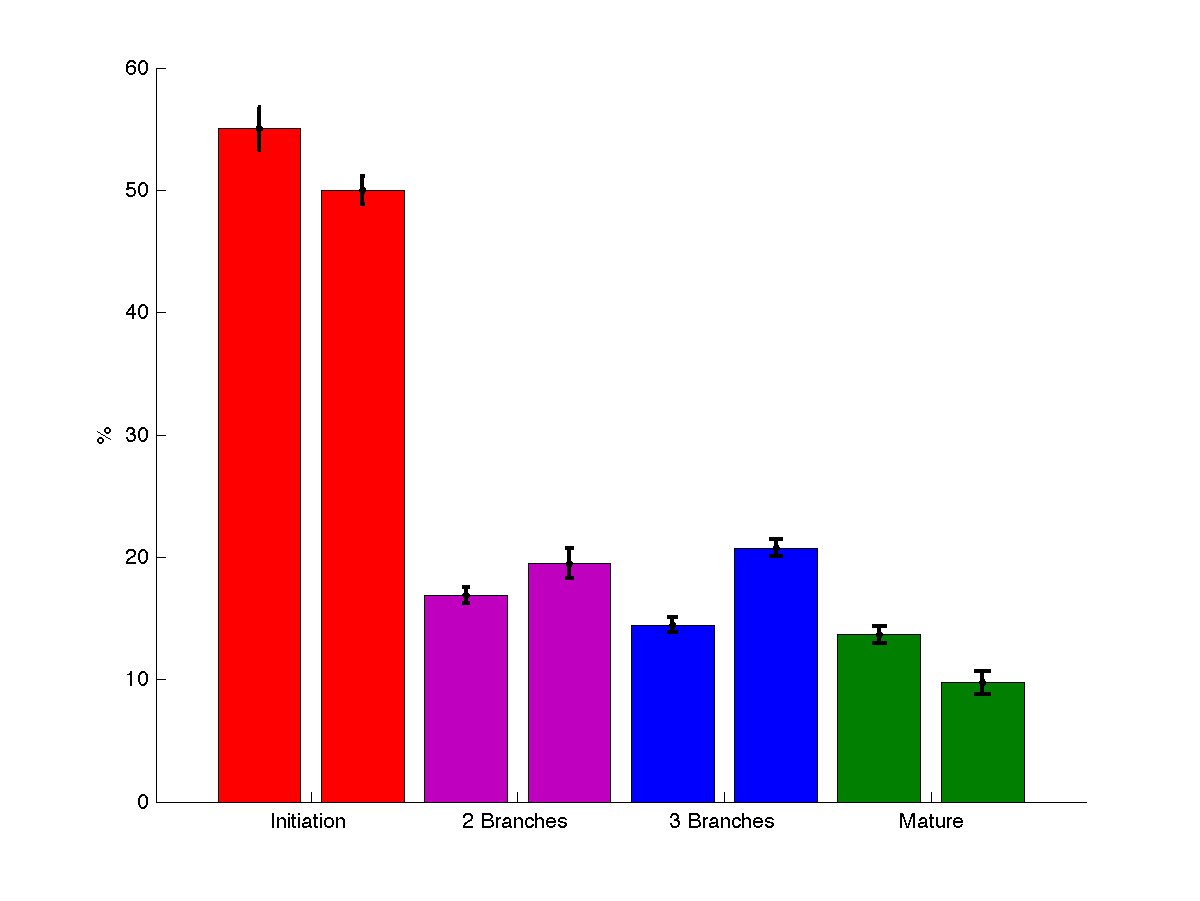

Supplement: Figure S1 — Relative abundance (in %) of trichome classes (red: initiation, magenta: 2-branch trichomes, blue: 3-branch trichomes, green: mature trichomes) for wildtype (left) and the cpc-2 mutant leaves (right). Error bars show standard deviations. (TIF) [file pcbi.1003029.s001.tif]

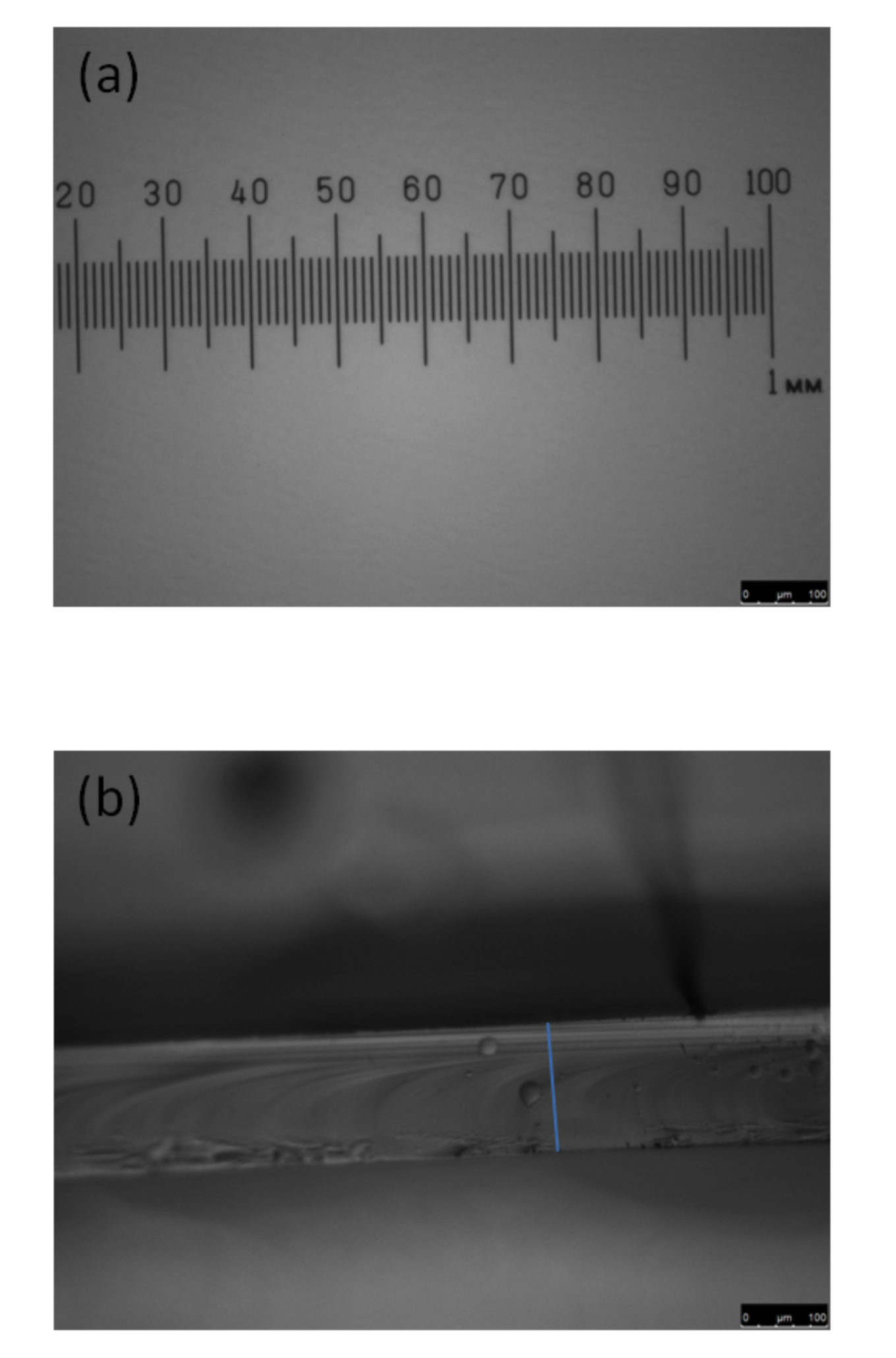

Supplement: Figure S2 — Microscopic pictures used to scale the z-axis. A: A scaling slide PYER SGI LIMITED (1 mm/0.01 mm DIV, Kent, UK) used to scale the 2D distance of the microscope. From that the ratio pixel per µm was determined. Each graduation equals 10 µm. B: A piece of a cover slide used for the z-axis scaling was placed vertically into agar. The line shows how the thickness of the cover slide was precisely measured. Subsequently, this piece of a cover slide was placed flat next to an acquired leaf and served as a reference for the z-axis. (TIF) [file pcbi.1003029.s002.tif]

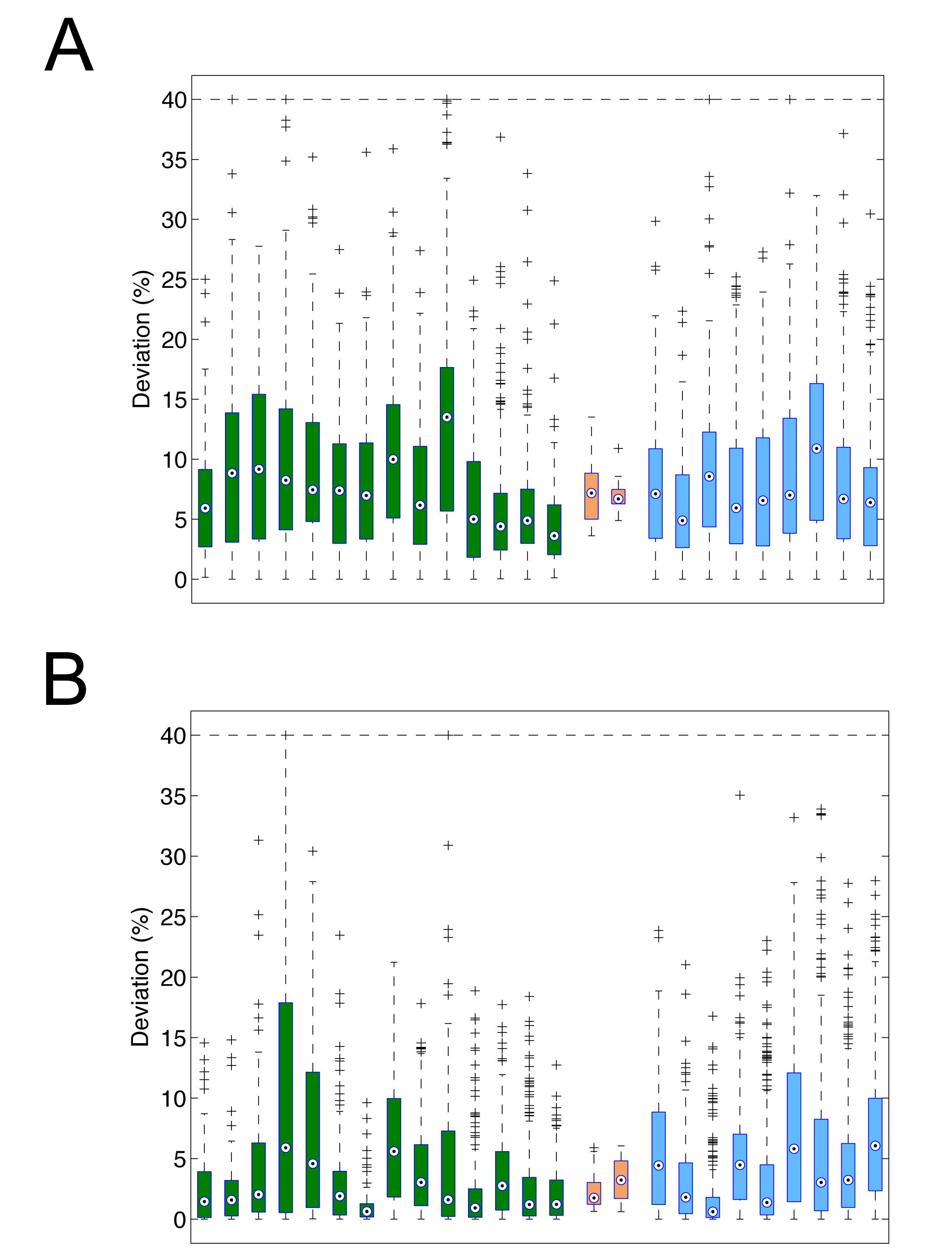

Supplement: Figure S3 — A: Relative increase (in %) of geodesic distances between two trichomes over the corresponding 3D Euclidean distances, summarized as separate boxplots for each leaf. Wildtype leafs are marked in green, leafs of cpc-2 are marked in blue. The orange boxes show the distribution of the median increases for wild type (left) and cpc-2 (right). B: Relative increase (in %) of 3D Euclidean distances between two trichomes over the corresponding 2D Euclidean distances. Coloring is as in A), identical leaves are shown in the same column as in A). (TIF) [file pcbi.1003029.s003.tif]

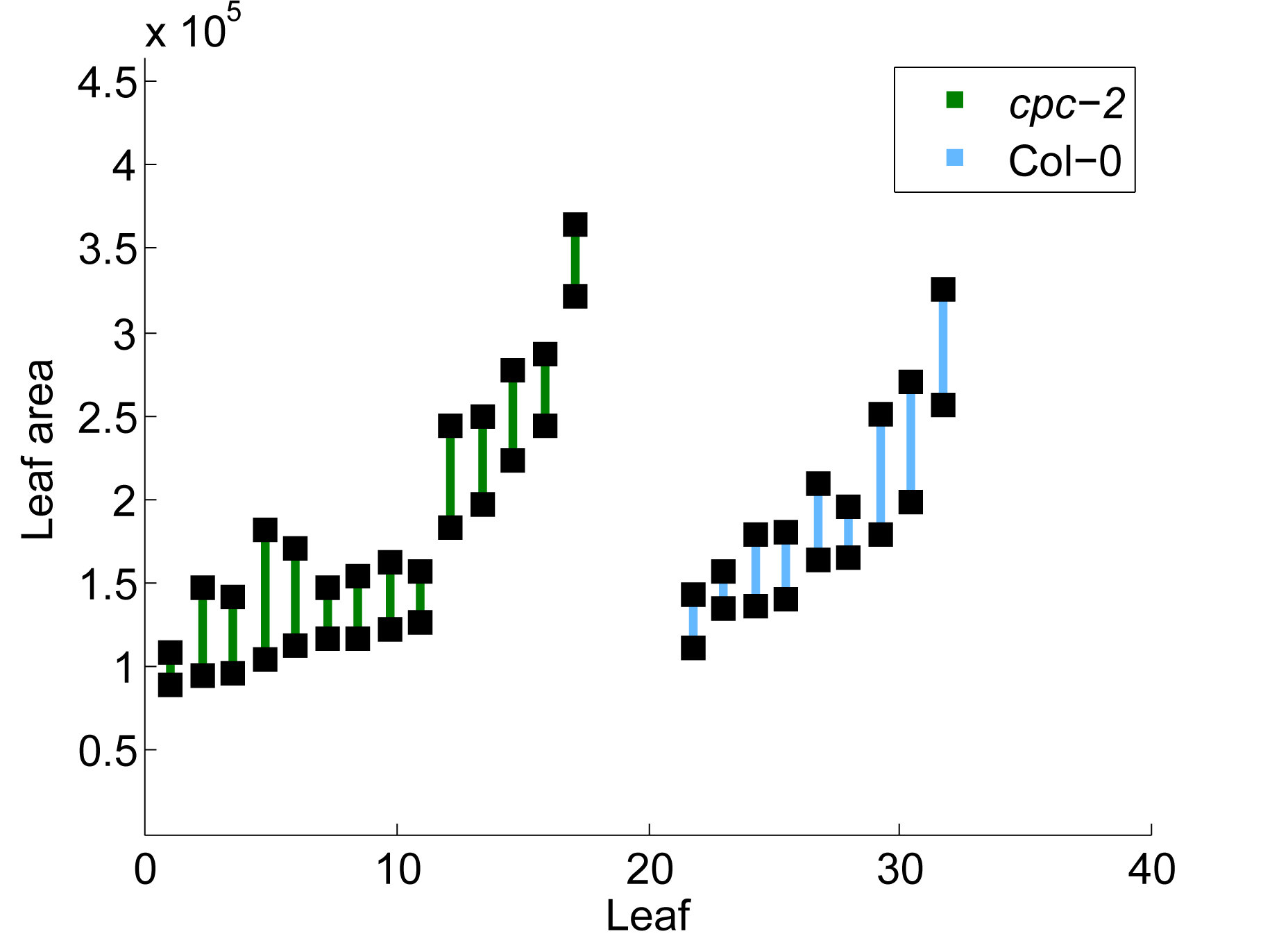

Supplement: Figure S4 — Comparison of 2D leaf area and 3D leaf area. Each line corresponds to one leaf (green: wild type, blue: cpc-2). The lower point of each vertical line corresponds to the 2D area, the upper point shows the 3D area. Leaf area is given in arbitrary units. (TIF) [file pcbi.1003029.s004.tif]

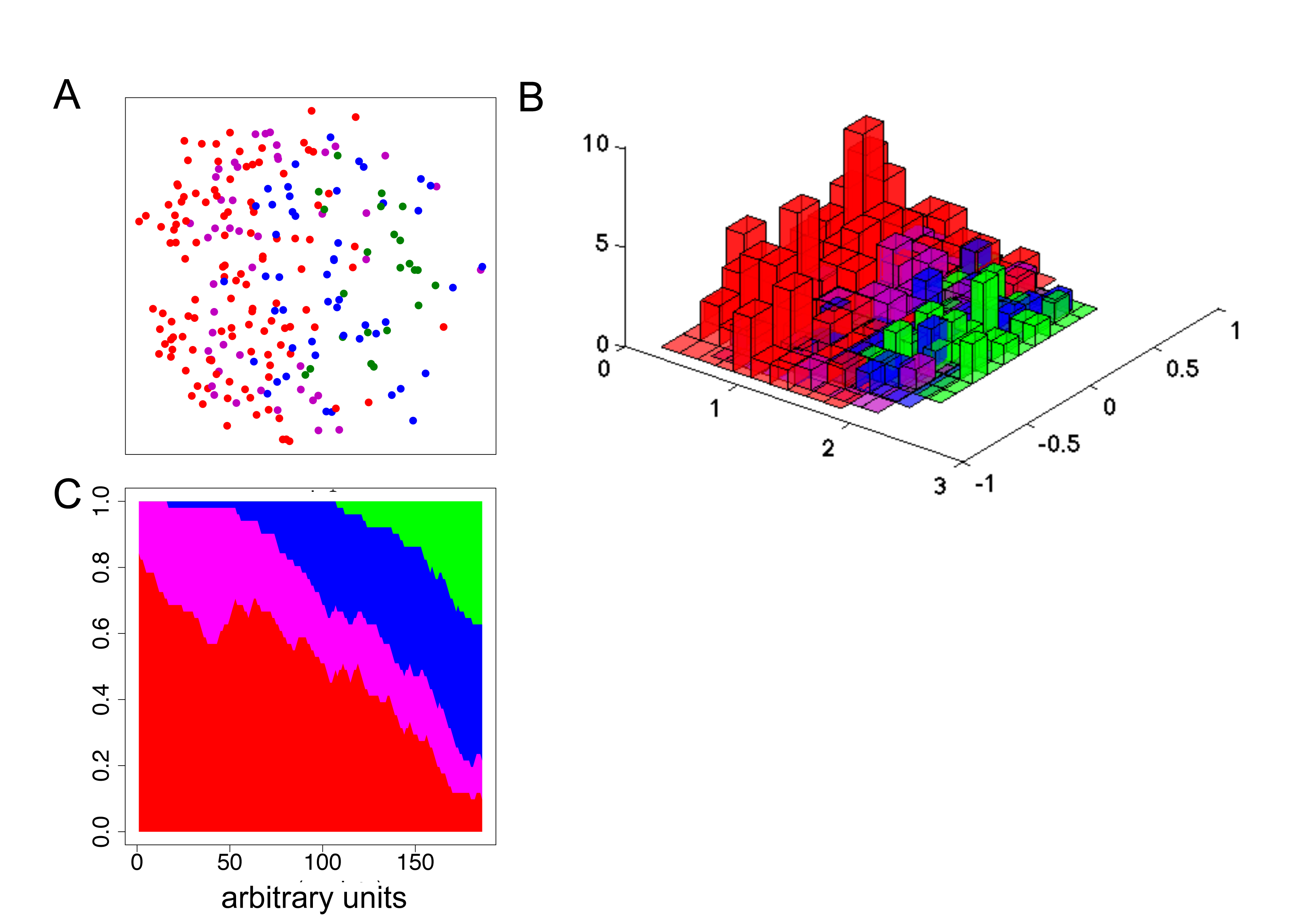

Supplement: Figure S5 — Meta leaf construction and analysis of spatial distribution of trichome classes for the cpc-2 genotype. A: The meta leaf is generated by transforming all trichomes of all leaves of a given genotype to a common coordinate system. The meta leaf shows the distribution of different trichome classes across the leaf, where red (respectively magenta, blue, green) dots indicate initiation (respectively two branch-, three branch-, and mature trichomes). B: The trichome localization along the longitudinal leaf axis is visualized. The vertical axis shows the proportion of different trichome classes at a given distance from the origin. C: The distribution of trichome classes on the meta leaf surface is shown in a 3D histogram. Trichome numbers at each position are shown as bars. (TIF) [file pcbi.1003029.s005.tif]

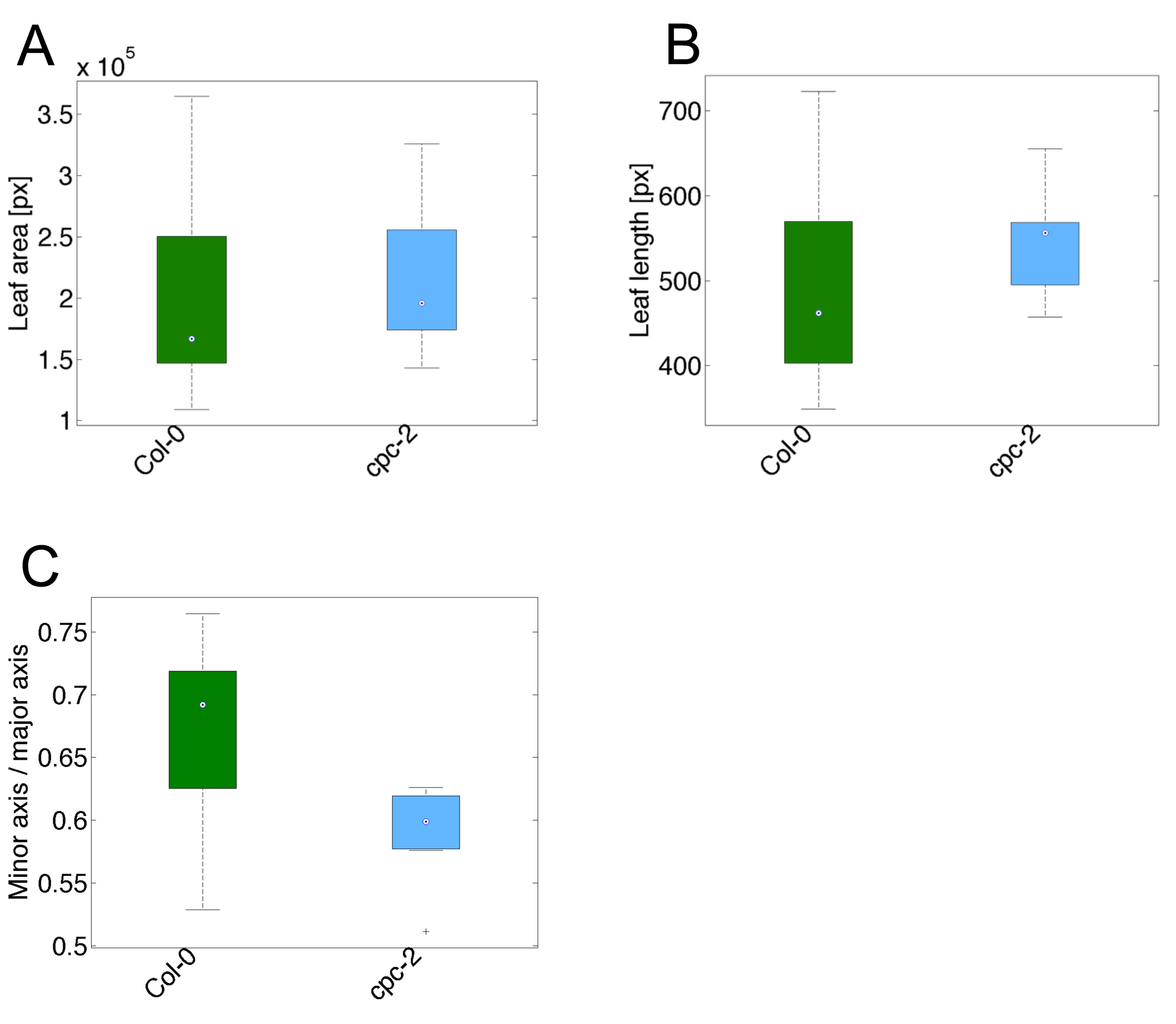

Supplement: Figure S6 — Comparison of leaf area, leaf length and leaf index for Col-0 and cpc-2. A: Leaf Area, given by the surface area of the elastic map. B: Leaf length, distance between leaf top and leaf base point. C: Leaf index, given by the quotient of leaf minor axis and leaf major axis. (TIF) [file pcbi.1003029.s006.tif]
