# Supplementary material for: Semi-automated 3D Leaf Reconstruction and Analysis of Trichome Patterning from Light Microscopic Images
Source: PLoS Comput Biol. 2013 Apr 18;9(4):e1003029. doi: 10.1371/journal.pcbi.1003029 (PMC3630213; doi:10.1371/journal.pcbi.1003029)
Supplement: Table S1 — Comparison between TrichEratops and other existing methods. Despite of all other methods TrichEratops combines light microscopy (without long sample preparation) and 3D reconstruction of the leaf surface. Furthermore it calculates patterning features similar to existing methods (voronoi area, metaleaf, coordinates). (DOCX) [file pcbi.1003029.s007.docx]

**Table S1**

| Features | Lee et al . 2006 [[1](#_ENREF_1)] | Kaminuma et al. 2008 [[2](#_ENREF_2)] | Bensch et al.2009 [[3](#_ENREF_3)] | Greese at al. 2012 [[4](#_ENREF_4)] | Pomeranz et al. 2012 [[5](#_ENREF_5)] | TrichEratops 2013 |
| --- | --- | --- | --- | --- | --- | --- |
| microscopy | Light microscope with optical projection tomography | µCT with xray detector | confocal laser microscopy | Light microscopy + confocal laser microscopy | Polarized light Microscopy | Light microscopy |
| Platform/program | QtVolView | Matlab | n.a. | Matlab + imageJ | Web browser + imageJ | Matlab |
| Preparation needed/time | Safranin staning, agarose embedding/ >3days | Dissection+mounting/unknown | GFP Marker line/ unknown | GFP Marker line/ unknown | Sterilisation + leaf clearing (~2day) | Dissection + mounting leaf on slide / 1 min |
| Marking trichomes | automatically | automatically | automatically | manually | Manually + automatic ally (20% error) | manually |
| Stage of leaves analyzed | Mature leaf | Mature leaf | Young leaf | Young leaf | Mature leaf | Mature and young leaf |
| 3D reconstruction | + | + | + | + | - | + |
| Output | Coordinates, density | Coordinates, density | Coordinates, density | Coordinates, density, voronoi area | Coordinates, density, heatmap | Coordinates, density, meta leaf, voronoi area |
| *Arabidopsis* lines tested | Wild type | Wild type, *cpc*, *gl3* | Wild type | Wild type, *try*, *gl1* | Wild type, *ttg2* | Wild type, *cpc* |

**Table S1:** Comparison between TrichEratops and other existing methods. Despite of all other methods TrichEratops combines light microscopy (without long sample preparation) and 3D reconstruction of the leaf surface. Furthermore it calculates patterning features similar to existing methods (voronoi area, metaleaf, coordinates).
