# Supplementary material for: Semi-automated 3D Leaf Reconstruction and Analysis of Trichome Patterning from Light Microscopic Images
Source: PLoS Comput Biol. 2013 Apr 18;9(4):e1003029. doi: 10.1371/journal.pcbi.1003029 (PMC3630213; doi:10.1371/journal.pcbi.1003029)
Supplement: Table S2 — Trichome counts for both genotypes and counts of finite Voronoi cells. Finite Voronoi cells are Voronoi regions that are bounded and whose edge points lie on the leaf surface. (DOCX) [file pcbi.1003029.s008.docx]

**Table S2**

| **Trichome counts** | | | | |
| --- | --- | --- | --- | --- |
|  | Initiation | 2 Branches | 3 Branches | Mature |
| Col-0 | 137 | 42 | 36 | 34 |
| *cpc-2* | 118 | 46 | 49 | 23 |
| **Finite voronoi cells** | | | | |
| Col-0 | 63 | 23 | 13 | 5 |
| *cpc-2* | 54 | 21 | 32 | 10 |

Table S2 Trichome counts for both genotypes and counts of finite Voronoi cells. Finite Voronoi cells are Voronoi regions that are bounded and whose edge points lie on the leaf surface.
