# Supplementary material for: Semi-automated 3D Leaf Reconstruction and Analysis of Trichome Patterning from Light Microscopic Images
Source: PLoS Comput Biol. 2013 Apr 18;9(4):e1003029. doi: 10.1371/journal.pcbi.1003029 (PMC3630213; doi:10.1371/journal.pcbi.1003029)
Supplement: Table S3 — Wilcoxon test for difference in trichome density between Col-0 and cpc-2. (DOCX) [file pcbi.1003029.s009.docx]

Table S3

|  | Initiation | 2 branches | 3 branches | Mature | All |
| --- | --- | --- | --- | --- | --- |
| p-value | 0.3610 | 0.0832 | 0.0107 | 0.5410 | 0.0791 |

Table S3. Wilcoxon test for difference in trichome density between Col-0 and *cpc-2*.
