# Supplementary material for: Semi-automated 3D Leaf Reconstruction and Analysis of Trichome Patterning from Light Microscopic Images
Source: PLoS Comput Biol. 2013 Apr 18;9(4):e1003029. doi: 10.1371/journal.pcbi.1003029 (PMC3630213; doi:10.1371/journal.pcbi.1003029)
Supplement: Table S5 — Wilcoxon test for difference in 3D Voronoi Area between Col-0 and cpc-2. (DOCX) [file pcbi.1003029.s011.docx]

Table S5

|  | Initiation | 2 branches | 3 branches | Mature | All |
| --- | --- | --- | --- | --- | --- |
| p-value | 0.0113 | 0.0031 | 0.0252 | 1 | 0.0034 |

**Table S5.** Wilcoxon test for difference in 3D Voronoi Area between Col-0 and *cpc-2*.
