# Supplementary material for: Semi-automated 3D Leaf Reconstruction and Analysis of Trichome Patterning from Light Microscopic Images
Source: PLoS Comput Biol. 2013 Apr 18;9(4):e1003029. doi: 10.1371/journal.pcbi.1003029 (PMC3630213; doi:10.1371/journal.pcbi.1003029)
Supplement: Table S6 — Wilcoxon test for difference in geodesic distance to nearest neighbor between Col-0 and cpc-2. (DOCX) [file pcbi.1003029.s012.docx]

Table S6

|  | Initiation | 2 branches | 3 branches | Mature | All |
| --- | --- | --- | --- | --- | --- |
| p-value | 0.0093 | 0.0011 | 0.12497 | 0.6429 | 0.0003 |

**Table S6.** Wilcoxon test for difference in geodesic distance to nearest neighbor between Col-0 and *cpc-2.*
