# Supplementary material for: Semi-automated 3D Leaf Reconstruction and Analysis of Trichome Patterning from Light Microscopic Images
Source: PLoS Comput Biol. 2013 Apr 18;9(4):e1003029. doi: 10.1371/journal.pcbi.1003029 (PMC3630213; doi:10.1371/journal.pcbi.1003029)
Supplement: Text S1 — Supplementary References. (DOCX) [file pcbi.1003029.s013.docx]

**References**

1. Lee K, Avondo J, Morrison H, Blot L, Stark M, et al. (2006) Visualizing plant development and gene expression in three dimensions using optical projection tomography. Plant Cell 18: 2145-2156.

2. Kaminuma E, Yoshizumi T, Wada T, Matsui M, Toyoda T (2008) Quantitative analysis of heterogeneous spatial distribution of Arabidopsis leaf trichomes using micro X-ray computed tomography. Plant J 56: 470-482.

3. Bensch R, Ronneberger O, Greese B, Fleck C, Wester K, et al. (2009) Image Analysis of Arabidopsis Trichome Patterning in 4d Confocal Datasets. 2009 Ieee International Symposium on Biomedical Imaging: From Nano to Macro, Vols 1 and 2: 742-745.

4. Greese B, Wester K, Bensch R, Ronneberger O, Timmer J, et al. (2012) Influence of cell-to-cell variability on spatial pattern formation. IET systems biology 6: 143-153.

5. Pomeranz M, Campbell J, Siegal-Gaskins D, Engelmeier J, Wilson T, et al. (2012) High-resolution computational imaging of leaf hair patterning using polarized light microscopy. Plant J.
